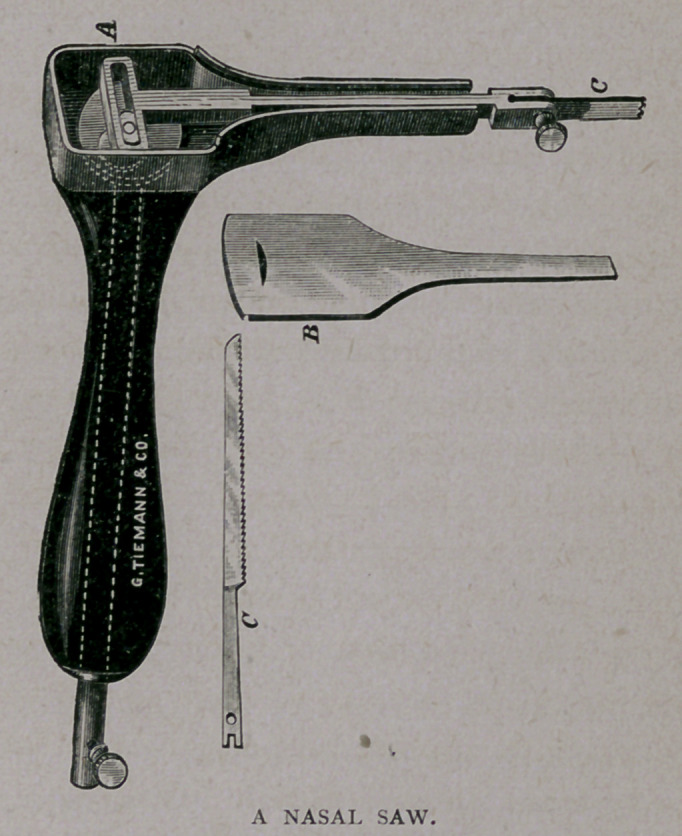# A Mechanical Nasal Saw

**Published:** 1889-06

**Authors:** Frank Hamilton Potter

**Affiliations:** Buffalo, N. Y., Lecturer on Diseases of the Nose and Throat, Medical Department, Niagara University; 273 Franklin Street


### New Instruments.

### A MECHANICAL NASAL SAW.

By FRANK HAMILTON POTTER, M. D., Buffalo, N. Y.,

Lecturer on Diseases of the Nose and Throat, Medical Department, Niagara University.

THE cut illustrates an instrument intended to overcome some of the difficulties in the use of the hand-saw in operations for removing hard obstructions from the nasal passages. Where the obstruction is sloping, it is often impossible to engage the ordinary saw just at the point desired, and, as a result, but part of it is removed. This neces-

A NASAL SAW.

sitates other operations. Again, when it is important to cut from below upward, the operator is at a disadvantage as to strength, and only succeeds with difficulty. The operator may enter this saw, even on a beveled surface, at the very point desired, and may also hold it in place without danger of slipping during the operation. By reference to the illustration it will be seen that it consists of a handle, through which runs a shaft, topped with a disk. The disk has a pin attached upon one side. As this disk is made to revolve, it carries backward and forward the saw-shaft by means of the arrangement shown below the letter *A*. The saw, *C*, is held firmly by a set-screw, and can be inserted with the teeth either up or down. The cover, *B*, fits securely upon the box, *A*, and thus protects the running parts from dust and injury. It is also easily removed for purposes of cleaning and oiling the parts. Special attention is called to the fact that, with the cover off, the saw-shaft can be lifted from its box, and this in turn allows the upright shaft to be pulled out of the handle ; first, of course, removing the set-screw seen at the lower end of this shaft. By this arrangement all the parts can be cleaned with very little trouble, and the entire apparatus rendered aseptic.

The instrument is light but strong, the handle being of hard rubber, and the other parts of metal. The power for running it may be either the dental engine or some of its modifications, or the electric motor. Whatever the power used, a flexible shaft, connecting it with the handle, will allow free movement to the hand of the operator and to the instrument, thus increasing the range of its usefulness.

Messrs. Tiemann & Co., of New York, have constructed the instrument, with attention to every detail, giving a most satisfactory result. Any order for it should be placed with them.

273 Franklin Street.

---

At the Hospital.—Surgeon—" What brought you to this dreadful condition? Were you run over by a street car?" Patient—" No, sir; I fainted and was brought to by a member of the 'Society of First Aid to the Injured.' "—*Life*. As this circumstance happened last Winter, it is probable the patient fainted in one of the Buffalo street cars, from breathing their foul air and sickening musty-hay odor.

---

How to Get Well.—When the doctors give you up, there is only one way to get well ; and that is to give the doctors up.—*Fliegende Blätter*.

---

The Erie County Medical Society will hold its semi-annual meeting on the second Tuesday in June—the 11th—at 10 o'clock a. m., in the Y. M. C. A. Building. There should be a full attendance.

## Figures and Tables

**Figure f1:**